# Supplementary material for: Cysteine cross-linking in native membranes establishes the transmembrane architecture of Ire1
Source: J Cell Biol. 2021 Jul 1;220(8):e202011078. doi: 10.1083/jcb.202011078 (PMC8256922; doi:10.1083/jcb.202011078)
Supplement: Table S1 — shows yeast strains used in this study. [file JCB_202011078_TableS1.docx]

Table S1. Yeast strains of used in this study

| Strain | Description | Genotype | Source |
| --- | --- | --- | --- |
| RE001 | BY4741 | ﻿BY4741 MATa; *his*3Δ1; *leu2*Δ0; *met15*Δ0; *ura3*Δ0 | Euroscarf |
| RE046 | Δ*IRE1* | ﻿BY4741 MATa; *his3*Δ1; *leu2*Δ0; *met15*Δ0; ura3Δ0; *ire1*Δ::kanMX4 | Euroscarf |
| RE127 | Δ*IRE1*Δ*IRE1promotor* | ﻿BY4741 MATa; *his3*Δ1; *leu2*Δ0; *met15*Δ0; *ura3*Δ0; *ire1*Δ::URA pUG72 | Halbleib *et al*. |
| RE425 | *IRE1*-3xHA-yeGFP | ﻿BY4741 MATa; *his3*Δ1; *leu2*Δ0; *met15*Δ0; *ura3*Δ0; *ire1*Δ::URA *IRE1*-3xHA-yeGFP::HIS pRE451 | Halbleib *et al*. |
| RE343 | *IRE1*-3xHA-yeGFP  *cysteine-less* | ﻿BY4741 MATa; *his3*Δ1; *leu2*Δ0; *met15*Δ0; *ura3*Δ0; *ire1*Δ::URA *IRE1*-3xHA-yeGFP::HIS pRE375 | This paper |
| RE342 | *IRE1*-3xHA-yeGFP C552 | ﻿BY4741 MATa; *his3*Δ1; *leu2*Δ0; *met15*Δ0; *ura3*Δ0; *ire1*Δ::URA *IRE1*-3xHA-yeGFP::HIS pRE374 | This paper |
| RE428 | *IRE1*-3xHA-yeGFP  W426A (IF2) | ﻿BY4741 MATa; *his3*Δ1; *leu2*Δ0; *met15*Δ0; *ura3*Δ0; *ire1*Δ::URA *IRE1*-3xHA-yeGFP::HIS pRE455 | Halbleib *et al*. |
| RE438 | *IRE1*-3xHA-yeGFP  T226A/F247A (IF1) | ﻿BY4741 MATa; *his3*Δ1; *leu2*Δ0; *met15*Δ0; *ura3*Δ0; *ire1*Δ::URA *IRE1*-3xHA-yeGFP::HIS pRE465 | Halbleib *et al*. |
| RE725 | *IRE1*-3xHA-yeGFP  cysteine-less  + CEN IRE1-3xFLAG-yeGFP *cysteine-less* | ﻿BY4741 MATa; *his3*Δ1; *leu2*Δ0; *met15*Δ0; *ura3*Δ0; *ire1*Δ::URA *IRE1*-3xHA-yeGFP::HIS pRE375 *IRE1*-3xFLAG-yeGFP::LEU pRE699 | This paper |
| RE726 | *IRE1*-3xHA-yeGFP C552 + *CEN* *IRE1*-3xFLAG-yeGFP C552 | ﻿BY4741 MATa; *his3*Δ1; *leu2*Δ0; *met15*Δ0; *ura3*Δ0; *ire1*Δ::URA *IRE1*-3xHA-yeGFP::HIS pRE374 *IRE1*-3xFLAG-yeGFP::LEU pRE700 | This paper |
| RE530 | *IRE1*-3xHA-yeGFP E540C *single cysteine* | ﻿BY4741 MATa; *his3*Δ1; *leu2*Δ0; *met15*Δ0; *ura3*Δ0; *ire1*Δ::URA *IRE1*-3xHA-yeGFP::HIS pRE575 | This paper |
| RE531 | *IRE1*-3xHA-yeGFP T541C *single cysteine* | ﻿BY4741 MATa; *his3*Δ1; *leu2*Δ0; *met15*Δ0; *ura3*Δ0; *ire1*Δ::URA *IRE1*-3xHA-yeGFP::HIS pRE576 | This paper |
| RE532 | *IRE1*-3xHA-yeGFP G542C *single cysteine* | ﻿BY4741 MATa; *his3*Δ1; *leu2*Δ0; *met15*Δ0; *ura3*Δ0; *ire1*Δ::URA *IRE1*-3xHA-yeGFP::HIS pRE577 | This paper |
| RE533 | *IRE1*-3xHA-yeGFP V543C *single cysteine* | ﻿BY4741 MATa; *his3*Δ1; *leu2*Δ0; *met15*Δ0; *ura3*Δ0; *ire1*Δ::URA *IRE1*-3xHA-yeGFP::HIS pRE578 | This paper |
| RE534 | *IRE1*-3xHA-yeGFP F544C *single cysteine* | ﻿BY4741 MATa; *his3*Δ1; *leu2*Δ0; *met15*Δ0; *ura3*Δ0; *ire1*Δ::URA *IRE1*-3xHA-yeGFP::HIS pRE579 | This paper |
| RE522 | *IRE1*-3xHA-yeGFP L545C *single cysteine* | ﻿BY4741 MATa; *his3*Δ1; *leu2*Δ0; *met15*Δ0; *ura3*Δ0; *ire1*Δ::URA *IRE1*-3xHA-yeGFP::HIS pRE570 | This paper |
| RE535 | *IRE1*-3xHA-yeGFP L546C *single cysteine* | ﻿BY4741 MATa; *his3*Δ1; *leu2*Δ0; *met15*Δ0; *ura3*Δ0; *ire1*Δ::URA *IRE1*-3xHA-yeGFP::HIS pRE581 | This paper |
| RE717 | *IRE1*-3xHA-yeGFP L547C *single cysteine* | ﻿BY4741 MATa; *his3*Δ1; *leu2*Δ0; *met15*Δ0; *ura3*Δ0; *ire1*Δ::URA *IRE1*-3xHA-yeGFP::HIS pRE691 | This paper |
| RE718 | *IRE1*-3xHA-yeGFP F548C *single cysteine* | ﻿BY4741 MATa; *his3*Δ1; *leu2*Δ0; *met15*Δ0; *ura3*Δ0; *ire1*Δ::URA *IRE1*-3xHA-yeGFP::HIS pRE692 | This paper |
| RE719 | *IRE1*-3xHA-yeGFP L549C *single cysteine* | ﻿BY4741 MATa; *his3*Δ1; *leu2*Δ0; *met15*Δ0; *ura3*Δ0; *ire1*Δ::URA *IRE1*-3xHA-yeGFP::HIS pRE693 | This paper |
| RE720 | *IRE1*-3xHA-yeGFP I550C *single cysteine* | ﻿BY4741 MATa; *his3*Δ1; *leu2*Δ0; *met15*Δ0; *ura3*Δ0; *ire1*Δ::URA *IRE1*-3xHA-yeGFP::HIS pRE694 | This paper |
| RE721 | *IRE1*-3xHA-yeGFP F551C *single cysteine* | ﻿BY4741 MATa; *his3*Δ1; *leu2*Δ0; *met15*Δ0; *ura3*Δ0; *ire1*Δ::URA *IRE1*-3xHA-yeGFP::HIS pRE695 | This paper |
| RE722 | *IRE1*-3xHA-yeGFP F544A C552  *single cysteine* | ﻿BY4741 MATa; *his3*Δ1; *leu2*Δ0; *met15*Δ0; *ura3*Δ0; *ire1*Δ::URA *IRE1*-3xHA-yeGFP::HIS pRE696 | This paper |
| RE723 | *IRE1*-3xHA-yeGFP F531R C552  *single cysteine* | ﻿BY4741 MATa; *his3*Δ1; *leu2*Δ0; *met15*Δ0; *ura3*Δ0; *ire1*Δ::URA *IRE1*-3xHA-yeGFP::HIS pRE698 | This paper |
| RE724 | *IRE1*-3xHA-yeGFP F531R F544C  *single cysteine* | ﻿BY4741 MATa; *his3*Δ1; *leu2*Δ0; *met15*Δ0; *ura3*Δ0; *ire1*Δ::URA *IRE1*-3xHA-yeGFP::HIS pRE697 | This paper |
| RE773 | *IRE1*-3xHA-yeGFP W426A E540C  *single cysteine* | ﻿BY4741 MATa; *his3*Δ1; *leu2*Δ0; *met15*Δ0; *ura3*Δ0; *ire1*Δ::URA *IRE1*-3xHA-yeGFP::HIS pRE575 | This paper |
| RE774 | *IRE1*-3xHA-yeGFP W426A T541C  *single cysteine* | ﻿BY4741 MATa; *his3*Δ1; *leu2*Δ0; *met15*Δ0; *ura3*Δ0; *ire1*Δ::URA *IRE1*-3xHA-yeGFP::HIS pRE576 | This paper |
| RE776 | *IRE1*-3xHA-yeGFP W426A F544C  *single cysteine* | ﻿BY4741 MATa; *his3*Δ1; *leu2*Δ0; *met15*Δ0; *ura3*Δ0; *ire1*Δ::URA *IRE1*-3xHA-yeGFP::HIS pRE579 | This paper |
| RE792 | *IRE1*-3xHA-yeGFP ER-dsRed-HDEL | BY4741 MATa; *his3*Δ1; *leu2*Δ0; *met15*Δ0; *ura3*Δ0; *ire1*Δ::URA *IRE1*-3xHA-yeGFP::HIS pRE451; Kar2sig.seq.-dsRed-HDEL::natR pRE850 | This paper |
| RE793 | *IRE1*-3xHA-yeGFP  *cysteine-less* ER-dsRed-HDEL | BY4741 MATa; *his3*Δ1; *leu2*Δ0; *met15*Δ0; *ura3*Δ0; *ire1*Δ::URA *IRE1*-3xHA-yeGFP::HIS pRE375; Kar2sig.seq.-dsRed-HDEL::natR pRE850 | This paper |
