# Supplementary material for: Cysteine cross-linking in native membranes establishes the transmembrane architecture of Ire1
Source: J Cell Biol. 2021 Jul 1;220(8):e202011078. doi: 10.1083/jcb.202011078 (PMC8256922; doi:10.1083/jcb.202011078)
Supplement: Table S2 — shows plasmids used in this study. [file JCB_202011078_TableS2.docx]

Table S2. Plasmids used in this study

| Plasmid | Description | Recombinant DNA | Source |
| --- | --- | --- | --- |
| pRE451 | *IRE1*-3xHA-yeGFP | pcDNA3.1  *IRE1*-3xHA-yeGFP WT | Halbleib *et al*. |
| pEv200 | pRS315 *IRE1*-yeGFP-HA | pRS315 *IRE1*-yeGFP-HA | van Anken *et al*. |
| pRE375 | *IRE1*-3xHA-yeGFP  *cysteine-less* | pcDNA3.1 *IRE1*-3xHA-yeGFP *cysteine-less* | This paper |
| pRE374 | *IRE1*-3xHA-yeGFP C552  *single cysteine* | pcDNA3.1 *IRE1*-3xHA-yeGFP C552 *single cysteine* | This paper |
| pRE455 | *IRE1*-3xHA-yeGFP W426A (IF2) | pcDNA3.1 *IRE1*-3xHA-yeGFP W426A (F2) | Halbleib *et al*. |
| pRE465 | *IRE1*-3xHA-yeGFP T226A/F247A (IF1) | pcDNA3.1 *IRE1*-3xHA-yeGFP  T226A/F247A (IF1) | Halbleib *et al*. |
| pRE699 | *CEN* *IRE1*-3xFLAG-yeGFP  *cysteine-less* | pRS315 *IRE1*-3xFLAG-yeGFP *cysteine-less* | This paper |
| pRE700 | *CEN* *IRE1*-3xFLAG-yeGFP C552  *single cysteine* | pRS315 *IRE1*-3xFLAG-yeGFP C552 *single cysteine* | This paper |
| pRE575 | *IRE1*-3xHA-yeGFP E540C  *single cysteine* | pcDNA3.1 *IRE1*-3xHA-yeGFP E540C *single cysteine* | This paper |
| pRE576 | *IRE1*-3xHA-yeGFP T541C  *single cysteine* | pcDNA3.1 *IRE1*-3xHA-yeGFP T541C *single cysteine* | This paper |
| pRE577 | *IRE1*-3xHA-yeGFP G542C  *single cysteine* | pcDNA3.1 *IRE1*-3xHA-yeGFP G542C *single cysteine* | This paper |
| pRE578 | *IRE1*-3xHA-yeGFP V543C  *single cysteine* | pcDNA3.1 *IRE1*-3xHA-yeGFP V543C *single cysteine* | This paper |
| pRE579 | *IRE1*-3xHA-yeGFP F544C  *single cysteine* | pcDNA3.1 *IRE1*-3xHA-yeGFP F544C *single cysteine* | This paper |
| pRE570 | *IRE1*-3xHA-yeGFP L545C  *single cysteine* | pcDNA3.1 *IRE1*-3xHA-yeGFP L545C *single cysteine* | This paper |
| pRE581 | *IRE1*-3xHA-yeGFP L546C  *single cysteine* | pcDNA3.1 *IRE1*-3xHA-yeGFP L546C *single cysteine* | This paper |
| pRE691 | *IRE1*-3xHA-yeGFP L547C  *single cysteine* | pcDNA3.1 *IRE1*-3xHA-yeGFP L547C *single cysteine* | This paper |
| pRE692 | *IRE1*-3xHA-yeGFP F548C  *single cysteine* | pcDNA3.1 *IRE1*-3xHA-yeGFP F548C *single cysteine* | This paper |
| pRE693 | *IRE1*-3xHA-yeGFP L549C  *single cysteine* | pcDNA3.1 *IRE1*-3xHA-yeGFP L549C *single cysteine* | This paper |
| pRE694 | *IRE1*-3xHA-yeGFP I550C  *single cysteine* | pcDNA3.1 *IRE1*-3xHA-yeGFP I550C *single cysteine* | This paper |
| pRE695 | *IRE1*-3xHA-yeGFP F551C  *single cysteine* | pcDNA3.1 *IRE1*-3xHA-yeGFP F551C *single cysteine* | This paper |
| pRE696 | *IRE1*-3xHA-yeGFP F544A C552  *single cysteine* | pcDNA3.1 *IRE1*-3xHA-yeGFP F544A C552 *single cysteine* | This paper |
| pRE697 | *IRE1*-3xHA-yeGFP F531R F544C  *single cysteine* | pcDNA3.1 *IRE1*-3xHA-yeGFP F531R F544C *single cysteine* | This paper |
| pRE698 | *IRE1*-3x-HA-yeGFP F531R C552  *single cysteine* | pcDNA3.1 *IRE1*-3xHA-yeGFP F531R C552 *single cysteine* | This paper |
| pRE789 | *IRE1*-3xHA-yeGFP W426A E540C  *single cysteine* | pcDNA3.1 *IRE1*-3xHA-yeGFP W426A E540C *single cysteine* | This paper |
| pRE790 | *IRE1*-3xHA-yeGFP W426A T541C  *single cysteine* | pcDNA3.1 *IRE1*-3xHA-yeGFP W426A T541C *single cysteine* | This paper |
| pRE793 | *IRE1*-3xHA-yeGFP W426A F544C  *single cysteine* | pcDNA3.1 *IRE1*-3xHA-yeGFP W426A F544C *single cysteine* | This paper |
| pSS455 | pRS303N TEF-Kar2signalsequence-dsRed-HDEL::natR (pRE850) | pRS303N TEF-Kar2signalsequence-dsRed-HDEL::natR | Sebastian Schuck  laboratory |
